# When color coding backfires: A guidance reversal effect when learning with realistic visualizations

**DOI:** 10.1007/s10639-021-10796-6

**Published:** 2021-11-09

**Authors:** Alexander Skulmowski

**Affiliations:** Digital Education, Institute for Informatics and Digital Education, Karlsruhe University of Education, Bismarckstr 10, 76133 Karlsruhe, Germany

**Keywords:** Visualization, Realism, Color coding, Cognitive load, Desirable difficulties

## Abstract

Digital learning increasingly makes use of realistic visualizations, although realism can be demanding for learners. Color coding is a popular way of helping learners understand visualizations and has been found to aid in learning with detailed visualizations. However, previous research has shown that color coding must not always be an effective aid, and that it even may reduce retention when used with simple visualizations. This study assessed whether the presence of color coding in learning tests has an effect after having learned using a detailed visualization that either featured color cues or one that did not. The results indicate that color coding helps learners the most if the learning tests also feature color coding. Importantly, learning with color-coded visualizations and being tested without color cues leads to the worst results in retention and transfer tests. Regarding transfer, color coding in the testing visualization boosts performance regardless of the presence of color cues in the learning phase. The results of this study challenge popular perspectives aiming at optimizing learning by removing potential sources of difficulty. Depending on the learning test, it may be more effective to keep a certain level of difficulty in the learning task when learning with digital media.

## Introduction

Learning with realistic visualizations is an important part of education and due the growing trend of digital learning, learners are destined to increasingly use detailed renderings and virtual models. However, realistic visualizations are known to impose a number of cognitive demands on learners. Working with detailed graphics requires a high level of spatial ability (Brucker et al., 2014; Huk, 2006) and is generally prone to inducing cognitive load (e.g., Scheiter et al., 2009). As a result, methods aimed at facilitating learning with realistic visualizations have been devised. For example, color coding has been examined as a tool to support learning using realistic visualizations and has indeed been shown to enhance learning in some studies (Dwyer, 1968; Skulmowski & Rey, 2018). The aim of this investigation is to assess whether color coding can also have negative effects when learning with realistic visualizations by preventing learners from generating a sufficiently stable mental representation.

### Color coding in visualizations: Blessing or curse?

Color coding is a ubiquitous design aspect of visualizations that has long been known to have benefits as well as potential negative consequences (Christ, 1975). In some studies, null or even negative effects of color coding on (search) performance and on learning were found. For instance, Christ (1975) summarizes multiple studies that revealed that color coding can even turn out to be distracting in a search task when elements are color-coded, but if participants were not informed of the color of the target element they had to identify. Furthermore, Christ describes that Egeth and Pachella (1969) hypothesized that color can be a dominant visual feature that takes attention away from other visual properties. However, Christ (1975) also highlights that the retention performance for color cues diminishes at a slower pace than for other visual features, such as shapes. It needs to be noted that the literature discussed in this paragraph until now is mainly concerned with search and identification tasks, and not with learning. Moving over to the field of learning and instruction, a study by Keller et al. (2006) that focused on presenting learners with rather abstract attributes concerning a construction site (such as the size of the area, the rate of return, and the number of issues), color coding did not result in a significant improvement, but only an advantage on the descriptive level. In sum, color coding cannot be considered to have a consistent advantage in tasks relying on rather abstract information.

Hegarty (2011) defines three types of visualizations of a varying degree of abstraction: (1) *iconic displays* which represent material objects and scenes, (2) *relational displays* such as scatterplots or Venn diagrams that represent abstract information, and (3) *hybrid displays*, combining iconic and relational displays. An example for the latter type would be a map with superimposed, color-coded temperature data, indicating the temperature of the different locations within the map (Hegarty, 2011). It has been shown that optimizing design choices of such hybrid displays that unite spatial information with displays of abstract properties can enhance task performance. For instance, presenting spatial data in the form of a map using color-coded overlays indicating temperatures or pressure levels, it has been found that increasing the visual saliency of relevant features after being instructed to work with meteorological maps can increase accuracy in assessing wind direction (Hegarty et al., 2010).

In the field of anatomy learning, color cues have been included in textbooks and other learning resources for a long time, thereby creating examples of the aforementioned hybrid displays. The results of color coding for this particular application appear more promising overall. For instance, Dwyer (1971) investigated the effects of color in heart anatomy visualizations. Dwyer’s predominant interest was whether color would make anatomical visualizations more attractive and therefore preferable for learning. Dwyer (1971) used colored and black-and-white versions of the following visualization types: schematic drawings, realistic drawings, photographs of a heart model, and photographs of the heart. In addition to these eight between-subjects groups, there was a control group that only listened to verbal instructions that all groups received. A number of different test such as identification tests, a drawing test, and terminology tests were used in that study. Color coding particularly helped participants in the identification test and total test scores. Related studies by Dwyer support the effectiveness of colors (Dwyer, 1968), show that color coding can particularly enhance learning for field-dependent learners (Dwyer & Moore, 1992), and provide evidence that color coding advantageous for female learners (Moore & Dwyer, 2001). Lamberski and Dwyer (1983) found out that color coding can be a beneficial way of guiding learners’ attention when instruction is paced by the learners themselves. A meta-analysis of Dwyer’s studies came to the conclusion that color has a positive effect on the learning performance of university students in terms of vocabulary learning, but not for secondary students (Reinwein & Huberdeau, 1997). Regarding text comprehension, the same meta-analysis concludes that color does not have a significant influence on learning performance (Reinwein & Huberdeau, 1997).

Color coding is often used as a means to visually cue the relations between parts of a visualization and other elements of learning material, such as text fragments, or to indicate category membership (e.g., Keller et al., 2006; Reisslein et al., 2014; Ozcelik et al., 2009; for a meta-analysis on signaling, see Richter et al., 2016). Jones (1962) highlights that color coding may not be particularly helpful for time critical tasks, but instead can contribute towards time savings in search tasks (see also Christ, 1975). Yeh and Wickens (2001) compared how map displays can be designed to maximize spatial information accessibility. They compared color coding with other design factors such as intensity coding (implemented through different brightness levels) and strategies aimed at reducing visual complexity (“decluttering”). Yeh and Wickens conclude that in their experiments, the primary function of color coding lies in segmenting a visual presentation, rather than in expressing relationships between elements. In a similar vein, the primary interest of the present investigation does not lie in the aspect of signifying relationships through color, but rather using color as a means to segment a visualization into visual chunks that are easier to distinguish. This strategy is commonly used in the field of medical education and does not necessarily require the accompanying label texts to be color-coded as well (e.g., Khalil et al., 2005). This type of segmentation using color coding has been shown to be an effective way of increasing learning performance when paired with realistic details in a study by Skulmowski and Rey (2018). In that study, a labeled bone model was presented in two levels of detail: as smooth, plain surface without details and as a highly detailed surface. Color coding was particularly helpful regarding the retention performance of the participants who learned with the more detailed rendering as indicated by an interaction effect of realism and color coding (Skulmowski & Rey, 2018). Importantly, color coding had an inverse effect on learning with the less detailed model in that study. With a low level of detail, participants achieved higher scores on the retention test when they did not receive a color-coded visualization. Furthermore, Skulmowski and Rey (2018) found a significant reduction of cognitive load through the use of color coding (see also Reisslein et al., 2014).

The summarized study suggests that learning tasks can be made to appear too easy for learners, potentially leading to worse performance as a result of learners not investing enough mental effort (Skulmowski & Rey, 2018). The authors link this result to the *disfluency effect* (e.g., Diemand-Yauman et al., 2011), which is a type of *desirable difficulty* (Bjork, 1994). Disfluency is a phenomenon in which making materials (perceptually) more difficult to process, for example by using a less legible font in a text, is believed to trigger attention, thereby creating the potential for a better learning performance (Diemand-Yauman et al., 2011; Seufert et al., 2017). Thus, disfluency belongs to the category of desirable difficulties, which refer to the aim of raising the difficulty of learning materials in order to foster learning (Bjork, 1994; Yue et al., 2013). However, as the findings surrounding the ideas of disfluency and desirable difficulties are generally presented as complex and somewhat inconsistent (Taylor et al., 2020; Weissgerber & Reinhard, 2017), warranting further investigation, in particular concerning the design of visualizations (Eitel et al., 2014).

In sum, the reviewed findings on color coding suggest that color cues can help learners to visually segment detailed visualizations. At the same time, color coding may lower the cognitive load involved in learning with realistic visualizations. However, the question arises whether a visual aid such as color cues can have the effect of making learners rely on these cues, potentially lowering their performance when being tested without cues.

### Aligning cognitive load and tests when learning with realistic visualizations

As summarized above, color coding can lower the cognitive load caused by realistic visualizations. Lowering cognitive load is one of the main recommendations that cognitive load theory (Sweller et al., 1998, 2019) gives for the design of instruction. It is assumed that given the limited working memory capacity of humans, learning can be optimized by avoiding any irrelevant cognitive load in order to give learners the opportunity to focus on the essential learning content (Sweller et al., 1998).

This strategy has recently been adapted to the specific challenges involved in digital learning. The approach of *cognitive load alignment* posits that cognitive load cannot be avoided in most instances of digital learning (Skulmowski & Xu, 2021). For perceptually rich media in particular, this strategy indicates that learning settings must be designed with an appropriate mode of testing in mind. Only if a suitable testing method is chosen, the additional cognitive load that may be required by forms of digital learning can be justified (Skulmowski & Xu, 2021). For instance, when using realistic visualizations, this approach suggests that the cognitive load caused by realistic details can only be acceptable if the learning objective includes knowledge of such visual details (Skulmowski & Xu, 2021). This assumption has been confirmed empirically. Skulmowski and Rey (2021) showed that the level of realism found in a test can alter the results after learners had learned using either a realistic or a schematic visualization. In that study, participants who had learned using the realistic visualization performed particularly well when they were tested using an equally detailed visualization. This has been referred to as a “concreteness-specific effect” on testing (Skulmowski & Rey, 2021), meaning that a matching degree of simplification in the learning and testing stages will lead to the best performance.

**Fig. 1:**
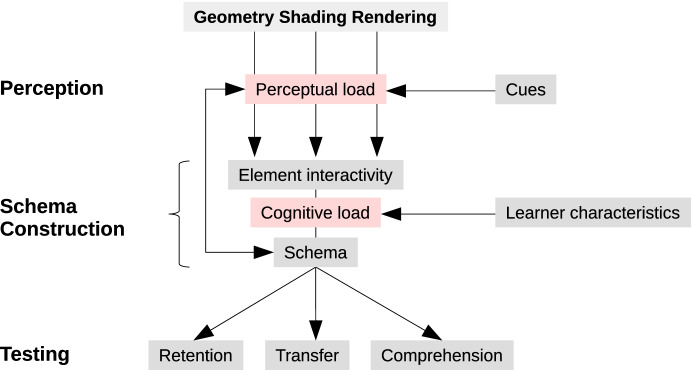
The *cognitive model of learning with realistic visualizations* by Skulmowski et al. (2021). In this model, learners view visualizations and need to process the perceptual load generated by the geometry, shading, and rendering. This perceptual load can be lowered through cues. The perceptual information is assembled into a mental schema and can then be tested using different types of tests. Reprinted with minor stylistic modifications from Skulmowski et al., (2021, p. 12), licensed under the Creative Commons Attribution 4.0 International License (http://creativecommons.org/licenses/by/4.0/), © 2021 Skulmowski, Nebel, Remmele, and Rey

The recently published *cognitive model of learning with realistic visualizations* (Skulmowski et al., 2021) synthesizes the findings of instructional realism research with a perspective informed by cognitive load theory and cognitive load alignment (see Fig. 1). In this model, learners are facing instructional visualizations whose level of realism is determined by the geometry, shading, and rendering of the virtual object(s) or scene. These parameters are thought to add to the perceptual load of a visualization (such as the number of details) which can, in turn, be made easier to process through cues such as color coding (Skulmowski et al., 2021). This model could be interpreted to suggest that color cues may be included in the mental schema that learners generate while memorizing the visualization. Thus, it may be hypothesized that learners could be overly reliant upon such cues during the later recall stage, suggesting a particular disadvantage for learners who were allowed to learn using cues, but are denied these cues during testing.

Taken together, color coding may be a method of lowering cognitive load during learning. However, there may be a danger in mismatching the learning task with the learning objective. If it is expected of learners to be able to have a mental representation of an object, letting them learn using color coding might be counterproductive, as they might assume that a later test will also include such cues.

### The present study

Based on the summarized literature, one might assume that learning with realistic visualizations can be facilitated using color cues. However, if learners are dependent on color coding to understand a detailed visualization, it seems plausible that learners who did not have the benefit of color coding during the learning stage may actually have an advantage if the testing materials also do not feature color cues. When learning without cues, learners may need to invest more mental effort to understand the visualization that could help them in a later test without cues. Hence, a guidance reversal effect can be assumed in which color coding will help learners if it is present both in the learning and testing materials, but leads to lower learning scores if the cues are only included in the learning stage. This effect was assumed for retention and transfer performance in the present experiment.

## Material and methods

### Participants and design

Based on previous research on realism and cues that found medium (e.g., η_p_^2^ = 0.06 in Skulmowski & Rey, 2018) to large effects (e.g., η_p_^2^ = 0.14 in Skulmowski & Rey, 2021, Exp. 1), a target sample size of 82 was determined based on an effect size of η_p_^2^ = 0.09 (power = 0.80) in a 2 × 2 between-subjects design using G*Power (Version 3.1.9.2; Faul et al., 2009). Thus, it was pre-determined that a sample of the first 82 participants would be used for the analyses. The aim was to stop the data collection as soon as these 82 participants had completed the study. Nine additional participants were able to complete the study before the data collection was stopped, but these datasets were not included in the final analyses. The participation criteria were that participants needed to be 18 to 30 years old native speakers of German using a PC or laptop. Participants could only take part once in the study. In order for a dataset to count as completed, three quality control questions needed to be answered appropriately; ensuring that participants took part in a conscientious manner and that they did not encounter technical difficulties or distractions (for more details on these questions, see below). Furthermore, they needed to have reached the final page of the web-based study. Regarding participants’ gender, 69 were female and 13 male. Participants were students at a German university of education enrolled in teacher training courses. They were students in a lecture on Digital Education open to students of all subjects at the undergraduate and graduate level. They took part for partial course credit.

Participants were assigned to four conditions using block randomization (in order to keep group sizes at a similar size) with the two factors color coding during learning (no color coding vs. color coding) and color coding during testing (no color coding vs. color coding). This design ensured that the effects of color coding during learning and color coding during testing could be assessed independently as well as in combination. Twenty-two participants completed the experiment with no color coding at all, 21 utilized color coding in the testing phase, 18 were able to learn using a color-coded visualization but did not get to use color coding during testing, and 21 participants were presented with color-coded visualizations in both the learning and testing stages.^1^

### Materials and procedure

**Fig. 2:**
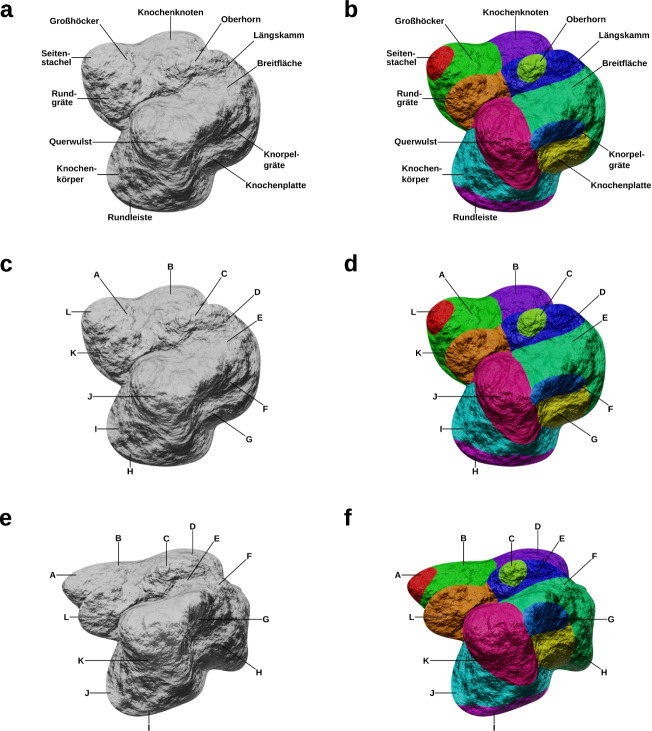
The bone visualization used in the experiment. Panels 2a and 2b show the models used in the learning phase, 2c and 2d contain the retention test, and 2e and 2f depict the transfer test. Panel 2a shows the bone model as it was displayed in the learning phase without color coding, 2b depicts the model presented to the groups who learned with color coding. Panels 2c and 2d feature the retention test without and with color coding, respectively. Panels 2e and 2f consist the transfer test without and with color coding, respectively. The presence or absence of color cues was kept constant during the retention and transfer tests. If participants were assigned to one of the groups who did not receive color cues during testing, this applied to both the retention and the transfer test

The study followed the method of Skulmowski and Rey (2018) in which renderings of a fictional bone model are presented to participants. The fictional bone used in this study (see Fig. 2) consists of 12 labeled components. The label texts were compound nouns generated by combining medical terms. Examples include labels such as “Knochenplatte” [bone plate], “Seitenstachel” [lateral spine], and “Großhöcker” [large tubercle]. Due to the fictional nature of the materials, a pre-test was not necessary (see Skulmowski & Rey, 2018). A procedural bump map was applied to the model in order to generate arbitrary details. Participants either viewed the version without (see Fig. 2a) or with color coding (see Fig. 2b). The retention and transfer tests (post-tests) were constructed as sorting tasks in which the 12 labels of the bone components needed to be assigned to the letters displayed in the test images (see Figs. 2c, d for the retention tests and Figs. 2e, f for the transfer tests). In both tests, either the retention or transfer test images were displayed, accompanied by an interactive sorting task. Participants needed to drag boxes labeled with component names on the corresponding slots marked with the letters found in the test visualizations. For each correctly assigned label, participants received one point on the tests. No additional tests, such as multiple-choice tests, were utilized. For the transfer tests, the original model was deformed using modifiers that twist and bend the geometry. The visualizations were created using Blender 2.91.2. The retention and transfer tests had a reliability of McDonald’s ω = 0.81 and ω = 0.84, respectively.

The study was conducted online using SoSci Survey Version (Leiner, 2021) which presented the different pages of the experiment using HTML and JavaScript. The experiment began with participants providing informed consent (including being informed about the fictional nature of the learning content). On the first page, they were asked to indicate whether they met the conditions for their data being included in the analysis (18 to 30 years old, German native speakers using a PC or laptop who did not already take part in the study). Following this page, the instructions were presented. Participants were informed that their task was to memorize the names, shapes and locations of the components of a bone presented on the following page. They were also instructed regarding the time limit of 60 s. In the learning phase, either the color-coded or non-color-coded model was presented together with a countdown timer. After the time had elapsed, participants were automatically directed to a filler task with a time limit of 60 s in which they were asked to sort the sixteen German federal states according to the number of their inhabitants. After the filler task, the retention and transfer tests followed on separate pages. On both pages, an instruction regarding the sorting task was given (labels presented on the pages should be sorted according to the letters presented in the test visualizations, see Fig. 2c-f). Participants were informed that there was no time limit for the tests and that they should not use any additional resources to answer the tests (similar tests have been used in Skulmowski & Rey, 2018, 2020, 2021). The presentation of color cues was kept constant during the retention and transfer tests. Participants who were assigned to one of the groups who were not presented with a color-coded visualization during the testing phase did not receive color cues during both the retention and the transfer test. After completing the tests, a page containing questions regarding participants’ gender and their course of study was presented. This page also featured quality control questions. Participants were asked whether they were strongly distracted during the learning task, whether there were significant technical difficulties, and whether they took part in the study conscientiously. Only if these three questions were answered appropriately, the dataset was considered complete and was used for further analyses. Finally, participants were thanked, given additional information on the study, and reminded that the bone presented in the study does not actually exist.

## Results

The analyses were performed using R (Version 4.0.5; R Core Team, 2021) with a significance level of 0.05.

### Retention scores

**Fig. 3:**
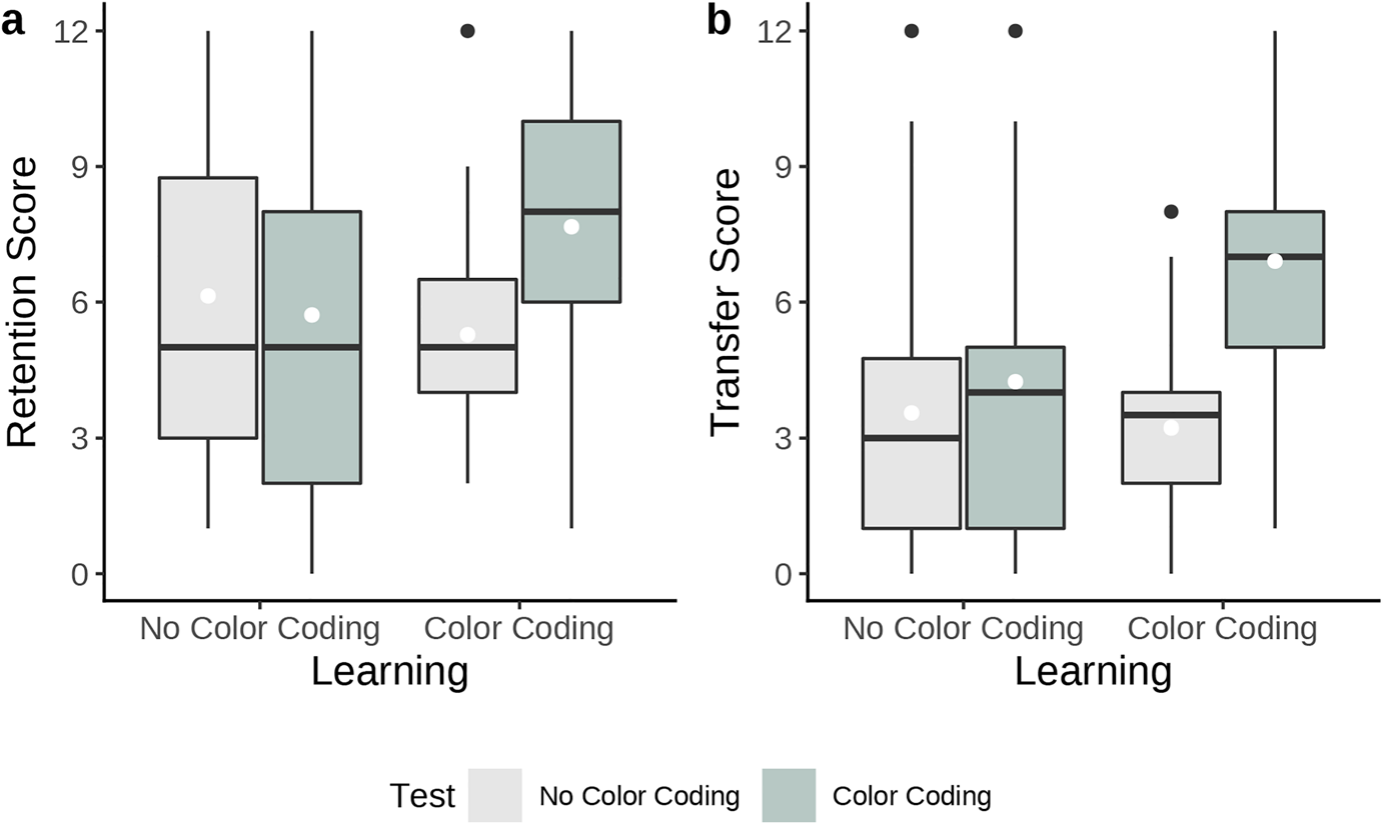
Boxplots of the untransformed data of the retention test (3a) and the transfer test (3b), both with a minimum of 0 points and a maximum of 12 points. White dots denote group means. In both boxplots, the two boxes on the left indicate the scores for the groups which learned without color coding, while the two boxes on the right show the scores for the groups which learned using color coding. The light gray boxes indicate which groups had to take both tests without color cues, while the light green boxes depict the scores of the groups that had access to the color-coded visualizations during testing

Corresponding to the 2 × 2 factorial design, analyses of variance (ANOVAs) were planned for the calculations of retention and transfer results. Participants’ retention scores were checked whether they conformed to the ANOVA assumptions of the normal distribution of the residuals and variance homogeneity. The Shapiro–Wilk test and Levene’s test did not reach significance, but approached this threshold (*p* = 0.052 and *p* = 0.097, respectively). Furthermore, the data of most groups exhibit a high variance with an *SD* of up to 3.8. As retention scores measured using similar tests have shown a tendency towards a high variance and outliers, robust ANOVAs with trimmed means (Wilcox, 2017) have been used in previous analyses (Skulmowski & Rey, 2020). An analysis with 10% trimmed means was chosen as a more suitable analysis method and revealed a significant interaction effect, value = 4.14, *p* = 0.047.^2^ The data shows that color coding has a divergent effect on retention performance (see Fig. 3a), with a strong advantage for those participants who were allowed to use color cues both during learning and testing (*M* = 7.67, *SD* = 3), and a disadvantage for participants who only used color coding during learning, but not during testing (*M* = 5.28, *SD* = 2.61). When no color coding was used during learning, the retention data even show a negative effect of color cues during testing (*M* = 5.71, *SD* = 3.54) compared to those who did not use color coding in either stage (*M* = 6.14, *SD* = 3.8), suggesting that a matched presentation format during learning and testing leads to the highest scores. The data indicate that learners fare best when cues that were present during learning are also available during testing. If this condition is not met, learners who were given cues during learning exhibit a marked drop in performance and achieve worse results than learners who were not given any cues at all. Consequently, the data support the hypothesized relationship between color coding in learning and testing.

### Transfer scores

The transfer data do not conform to the assumption of residual normal distribution as indicated by a significant Shapiro–Wilk test (*p* < 0.001). Hence, an ANOVA using aligned rank transformation (Fawcett & Salter, 1984) was selected for the analysis. The hypothesized interaction effect reached significance, *F*(1, 78) = 5.82, *p* = 0.018, η_p_^2^ = 0.07.^3^ Similarly to the result pattern in the retention test, participants who had learned using the color-coded visualization and were tested without these cues scored lowest in the transfer test, *M* = 3.22, *SD* = 2.16 (see Fig. 3b). Learning with color cues and being tested using them elicited the highest scores (*M* = 6.91, *SD* = 3.18). In contrast to the retention test, being tested with color cues increased scores even if participants had not learned using color coding (*M* = 3.55, *SD* = 3.35 without cues during testing; *M* = 4.24, *SD* = 3.55 with cues during testing). Thus, in the transfer test, the presence of color cues in the testing stage significantly increased scores regardless of whether learners had access to them previously, *F*(1, 78) = 10.78, *p* = 0.002, η_p_^2^ = 0.12. Due to the interaction effect, another significant main effect for the comparison between the use of color coding during learning cannot be interpreted, *F*(1, 78) = 5.70, *p* = 0.019, η_p_^2^ = 0.07. As for the retention data, the transfer scores show that learners achieve the best scores when cues that were given during learning are also presented during testing. Again, if learners who were given cues during learning do not have access to cues during testing, they end up with lower scores than learners who did not get to see any cues during both phases. This pattern is even more pronounced for the transfer data compared with the retention results and supports the hypothesized guidance reversal effect of color cues when learning with realistic visualizations.

## Discussion

Facilitating learning with realistic visualizations through color cues can be detrimental to learning for tasks in which the later testing does not include these color cues. As learners typically need to apply knowledge gained from visualizations to real-world situations that do not contain such cues, the results suggest that the use of cues may hinder performance, in particular when this knowledge needs to be transferred. However, including cues in tests appears to raise performance in transfer tests while neither having a positive nor negative effect on retention performance.

The study suggests that offering learners guidance may not necessarily have only positive consequences. In fact, learners might become reliant on such cues, leading them to struggle when cues available in the learning phase are not included in learning tests. This result pattern was shown both in the retention and the transfer test. One explanation for this effect is that learners might rely on the boundaries provided by the color cues during learning and then are ill-prepared for tests without these cues. As the highest scores were reached in both tests when the color cues were presented both in the learning and testing stage, learners might integrate some of the color information into their mental schema, aiding later information retrieval.

Interestingly, the retention and transfer results differ in one important aspect. While the use of color coding during testing regardless of the presence of color cues during learning did not improve retention performance overall, displaying color cues helped all learners in the transfer test. This difference is a possible hint at different functions that color cues can have in different types of tests. For a retention test in which the mere retrieval of information is the essential objective, it might not make a substantial difference whether color coding is available at the testing stage if learners did not have this color coding present during the learning phase. For the mere retrieval of components in a visualization, learners can resort to various learning strategies and mnemonic devices and, thus, are not completely dependent on this one particular type of cue. However, as soon as the shape of the model is transformed for the transfer task, color coding, even if it is only available during the testing stage, can make a valuable contribution towards understanding how the geometry of the model was deformed (see Skulmowski et al., 2021, for a discussion of the different components of realistic visualizations). As such a type of transfer task requires information retrieval *and* the correct application of this knowledge to a modified visualization, the latter component of this task may be the underlying cause why the inclusion of color cues boosted performance in the test regardless of the use of color coding in the learning task. In this task, color coding may have acted as a guide facilitating learners’ understanding of the changes in the model from the original bone presented during learning.

## Implications for the design of visualizations

The results suggest that learning with realistic visualizations can actually benefit from avoiding a (well-meant) simplification depending on the learning test. Thus, the study is an example of how guidance can have a negative effect on learning under certain circumstances. These results are in line with the idea of desirable difficulties, suggesting that making learning too easy for learners can turn out to be detrimental. Furthermore, the findings support the strategy of cognitive load alignment (Skulmowski & Xu, 2021). While it may be possible to lower cognitive load using color coding, the mental effort that learners need to invest while mentally processing a complex visualization can actually be thought of as an important component of the learning task if the learning test does not offer such cues. Therefore, the otherwise unnecessary cognitive load of having to deal with a complex realistic visualization becomes an intrinsic part of the task that should not be avoided (see Skulmowski & Xu, 2021). More generally, the results confirm that learning tests must be carefully chosen for perceptually rich forms of digital media (see Skulmowski & Xu, 2021).

The findings underline that facilitating learning must not always be the optimal strategy if a task requires learners to be prepared to work without cues. Hence, more research should be conducted whether other methods of reducing cognitive load during learning can similarly lead to guidance reversal effects. However, the results do not indicate that color coding should generally be avoided. Rather, the specific function at distinct points in a learning task should be considered more strongly. For example, the reduction in cognitive load brought about by color coding may be an effective way of providing learners with an initial understanding of complex visualizations. Following the strategy of guidance fading (Renkl & Atkinson, 2003), color coding might turn out to be a highly valuable tool at the beginning of a learning task that may work best if it is dropped over the course of that task. Using such a strategy, the advantages of color coding could be achieved without the drawback of a reliance of cues. Further research is needed to assess whether guidance fading can have a positive effect on learning with color-coded realistic visualizations. In particular, it should be determined how the effects discussed affect learning in more extensive scenarios such as immersive virtual worlds (see Albus et al., 2021, for an example of using text-based signals in virtual reality).

## Limitations

The study used a highly controlled method of presenting realistic visualizations while avoiding the influence of differences in prior knowledge. At the same time, the study was conducted online to achieve ecologically valid insights into the learning process with realistic visualizations and color coding. Future research should evaluate whether the effects found in the study can be replicated with (specific types of) nonfictional learning materials and whether a laboratory setting influences the result pattern. However, given the potential for future closures of schools and universities in the ongoing COVID-19 pandemic, additional online studies conducted using controlled methods will be important contributions as well.

Previous research has indicated that spatial ability plays an important role during learning with realistic visualizations (e.g., Huk, 2006). As the present study did not allow participants to freely rotate or move the models, this aspect may have exerted a less strong influence, but could be explored in follow-up studies, particularly in virtual reality. Furthermore, the study did not take into account forms of color blindness and did not check for color vision deficiencies in participants. Even if participants with such decreased abilities took part, the differences in value found in the different colors of the visualizations will still have fulfilled the desired function of visually segmenting the model.

## Conclusion

Color coding can facilitate learning by making complex visualizations easier to comprehend, thereby lowering cognitive load. This study has shown that it is possible to simplify learning in a harmful way as soon as a test does not provide color cues. While learning with detailed visualizations can be demanding, it is important not to “optimize” learning at the expense of creating problems for learners later on. As learning tasks should be constructed with the later application of the learned knowledge in mind, color coding in the context of realistic visualizations should not be used in a manner that makes learners reliant upon these cues. Taken together, the paper highlights the importance of more research on the effects of reducing cognitive load in visualizations and how to prevent such a strategy from backfiring.

## Data Availability

The data of this study are available from the author upon request.

## Footnotes

1 Minor differences in group sizes occurred despite block randomization due to participants not completing the experiment.

2 A parametric ANOVA did not result in a significant interaction effect, *p* = 0.058. The inclusion of the nine additional participants that were able to complete the study after the target of 82 participants was reached also prevents this effect from reaching significance using an ANOVA with aligned rank transformation (see Sect. 3.2), *p* = 0.134.

3 This interaction effect also reaches significance when analyzed using the 10% trimmed means approach used for the retention data, value = 4.22, *p* = 0.045. All effects on transfer that are significant in the sample of 82 participants reported in this section remain significant when the additional nine participants who completed the experiment after the target sample size had been reached are included in the analysis (using an ANOVA with aligned rank transformation).
